# Self-Inflicted Gun Shot Wounds: A Retrospective, Observational Study of U.S. Trauma Centers

**DOI:** 10.5811/westjem.2021.4.49315

**Published:** 2021-05-19

**Authors:** Faith Quenzer, Andrew Givner, Rachel Dirks, Christopher J. Coyne, Frank Ercoli, Ricard Townsend

**Affiliations:** * University of California, San Diego, Department of Emergency Medicine, San Diego, California; † Desert Regional Medical Center, Department of Emergency Medicine, Palm Springs, California; ‡ University of California, San Francisco-Fresno, Department of Emergency Medicine, Fresno, California; § Desert Regional Medical Center, Desert Trauma Surgeons, Palm Springs, California

## Abstract

**Introduction:**

Intentional self-harm (suicide) by firearms is a growing problem in the United States. Currently, there are no large studies that have identified risk factors for patients who die from self-inflicted gunshot wounds. Our objectives are to 1) identify risk factors for patients with the highest morbidity and mortality from self-inflicted gunshot wounds (SIGSWs) at trauma centers 2) present the outcomes of victims of SIGSW by handguns (HG) versus all other specified guns (AOG) and 3) compare the presentations and outcomes of victims with head or face (HF) injuries to other regions of the body.

**Methods:**

We performed a retrospective analysis from the National Trauma Database (NTDB) data between 2012 and 2013 of all SIGSW patients who presented to trauma centers. Categorical data included patient characteristics upon presentation and outcomes which were compared between patients with HG injury versus AOG injury using the Chi-Squared test, where AOG includes shotguns, hunting rifles, and military firearms. Additionally, analysis of head and face (HF) injuries versus other bodily injuries (OBI) were compared between the HG group versus AOG group using Chi-squared test.

**Results:**

There were 7,828 SIGSWs, of those, 78% (6,115) were white and 84.3% (6,600) were male. There were 5,139 HG injuries, 1,130 AOG injuries, and 1,405 unidentified gun injuries. The HG group was likely to be older (>55 years old), hypotensive (systolic blood pressure < 90), have a lower Glasgow Coma Score (GCS < 9), use illegal, or use prescription drugs. In comparing HF injuries (4,799) versus other bodily injuries (OBI) (3,028), HF group was more likely to use handguns, expire in ED, require ICU, and have a higher percent of overall mortality. Of the total OBI, the thorax, upper extremities, and abdomen were the most commonly injured.

**Conclusion:**

In our retrospective study of SIGSWs, we were able to demonstrate that SIGSW by handguns are associated with higher rates of mortality versus all other types of firearms. SIGSWs in older white males with handguns are the most at-risk for severe complications. Future efforts should improve screening methods for handguns in suicidal patients and at developing prevention programs.

## INTRODUCTION

Intentional self-harm (suicide) is a growing problem in the United States (U.S.) and has recently become one of the top ten leading causes of death. Earlier studies have shown that higher rates of firearm ownership are strongly associated with higher rates of firearm suicide.^1^–^2^ We designed this study to investigate several characteristics surrounding self-inflicted gunshot wounds (SIGSWs) that present to designated trauma centers.

According to the most recent data in 2017, a total of 39,773 deaths were due to firearms, which has increased since the previous year. Reportedly, 60% of these firearm arm deaths were self-inflicted. Whereas, firearm deaths due to assault accounted for 36.6%. Despite the fact that a majority of firearm deaths were self-inflicted, there is still a limited amount of research and data on self-inflicted firearm deaths and injury.^1^–^3^ Additionally, self-inflicted gunshot wounds are not always clearly defined as intentional, as in suicide. For the purpose of our study, self-inflicted gunshot wound (SIGSW) is defined as a gunshot wound while the gun was in the possession of the injured person at the time of firing, with an unknown intent of the shooter. Conversely, assault by gunshot wound will be defined as when the gun was not in the possession of the injured person at the time of firing.

Our objective is to compare the presentations and outcomes of victims of self-inflicted gunshot wounds (SIGSW) by handguns (HG) versus all other specified guns (AOG) group. Additionally, we compare the presentations and outcomes of victims with head or facial (HF) injuries to those with injuries to other regions of the body.

## METHODS

We performed a retrospective analysis of data, which was taken from the National Trauma Database (NTDB). This data represents all patients of all ages who presented to designated trauma centers in the United States (U.S.) between 2012 and 2013. The data were extracted from various external cause of injury codes (e-codes). These e-codes were diagnosis codes to explain the circumstances and the external causes of a particular injury prior to the use *International Classification of Diseases, Tenth Revision, Clinical Modification* (ICD-10-CM) codes. Patients who presented to designated U.S. trauma centers with e-codes 955.0 (Suicide and self-inflicted injury by handguns), 955.1 (Suicide and self-inflicted injury by shotgun), 955.2 (Suicide and self-inflicted injury by hunting rifle), and 955.3 (Suicide and self-inflicted injury by military firearm) were included in the analysis. From the e-codes, patient demographics, characteristics, and outcomes were analyzed by using contingency tables and the Chi-Square test. We compared the characteristics and presentations of those who sustained a HG injury versus AOG. Any firearm that was not a HG was an AOG. These AOGs include shotguns, hunting rifles, and military firearms. Additionally, a subgroup analysis was performed which compared head and face (HF) injuries versus other bodily injuries (OBI) using the Chi-squared test.

## RESULTS

From the National Trauma Database (NTDB), a total of 7,828 cases of SIGSWs presented at designated U.S. trauma centers from 2012 to 2013. Of these SIGSWs, there 5,139 HG injuries and 1,130 AOG injuries. The raw data show that males accounted for 6,600 (84.3%) patients and females accounted for 1,228 (15.7%) patients. Of the total number of SIGSWs, 6,115 (78%) were identified as White. There were 1,405 SIGSWs that were excluded from the analysis because the data did not identify the type of firearm involved. Additionally, 154 patients whose injuries may not have been a SIGSW were excluded from analysis.

**Population Health Research Capsule**

What do we already know about this issue?

*Suicide by firearms is a growing problem in the U.S.*

What was the research question?

*What factors increase the morbidity and mortality of self-inflicted gunshot wounds (SIGSWs) at trauma centers?*

What was the major finding of the study?

*Handguns are associated with higher morbidity and mortality in SIGSWs in older white males.*

How does this improve population health?

*Our study highlights the need to screen suicidal patients with firearm access in the emergency department.*

In comparing the two SIGSW groups; patients who sustained HG injuries were more severely injured compared to AOG injuries. As observed in Table 1, patients in the younger than 55-year-old age group who sustained SIGWS were more likely to use all other guns (shotguns, hunting rifle, military firearms). The HG group was more likely to be older than 55 years of age (p < 0.001), male (p = 0.001), and hypotensive with systolic blood pressure less than 90 mmHg (p < 0.001). The HG group was also more likely to have a Glasgow Coma Score (GCS) less than 9 (p < 0.001). In those with a GCS total of 9 to 13, there was no statistical difference between the HG and the AOG groups. However, the AOG patients were more likely to have a GCS of 14 or 15 (51%) versus the HG group (39%) with p < 0.001.

There was no difference between the proportion of those who tested positively for alcohol intoxication in the HG versus AOG groups (p = 0.25). The 1,581 (49%) patients in the HG group and 367 (51%) patients in the AOG group tested positively for alcohol. Only a limited number of patients received toxicology panels: 2,013 of the HG group and 438 of the AOG group. Among these groups, the HG group had a higher proportion of patients who tested positively for illicit drugs versus 740 (37%) versus the AOG group 129 (30%) (p=0.004). While interestingly, the AOG group had a higher proportion of who tested positively for prescription drugs with 212 (48%) versus the HG group with 719 (36%), p<0.001.

In examining SIGSW bodily injuries, head or facial (HF) injuries were more lethal and presented with severe morbidity compared to other bodily injuries (OBI) in the ED. In Table 2, of the 4,799 HF injuries, 1,052 (22%) resulted death in the ED versus the 111 (4%) of the 3,028 OBI patients (p < 0.001). Of the HF injured patients, 2,768 (58%) required ICU care versus 531 (18%) of the OBI patients (p < 0.001). Those with OBI injuries were more frequently admitted to the hospital floor 660 (22%), taken to the OR 1,303 (43%), or discharged home versus their HF injured counterparts with p <0.001. Of the HF injured patients, 2,817/4,799 (59%) died during their presentation to the ED versus 365/3,048 (12%) of the OBI patients (p < 0.001).

The categorization of the 2012 to 2013 NTDB data follows a trimodal model supported by earlier trauma where severity of injury is categorized trauma associated mortality.^4^ Immediate death or dead-on-arrival (DOA) occurs within minutes to within an hour of arrival at the hospital. These patients are likely to have sustained unsurvivable injuries. Additionally, patients who die within the four-hour interval are also likely to have sustained serious, severe injuries but will take into account for regional transport time from the trauma scene and to the hospital trauma center.^4^ Those who die within the 4-to-24-hour time frame also have but are considered to have been potentially treatable with prompt definitive care.^4^,^5^ Those who die within the 24–72-hour timeframe also has treatable injuries, but likely die from complications of the inciting trauma.^4^ Those who die outside of the 72-hour time frame, likely die from a complication other than the trauma itself such as pulmonary embolisms.^6^

We compared several time of death intervals between the HF and OBI groups. There was no statistically significant difference between those who presented DOA to the ED between the two groups. In the OBI group, a greater proportion died within four hours of arrival and in the greater than 72 hours versus the HG group (p < 0.001). A greater proportion of the HG group died within the 4–24 hour time frame and the 24–72 hour time frame than the AOG group (p < 0.001).

In the subgroup analysis of the 3,028 OBI, the most common region of the body injured was the thorax 1,261 (42%), followed by 924 (30%) upper extremity injuries, 885 (29%) abdominal injuries, and 783 (26%) lower extremity injuries. There were only 118 (4%) SIGSW patients who presented with spinal injuries. In those categorized with HF injuries in the ED, 252 (5%) presented with neck injuries versus OBI with 114 (4%) (p = 0.002).

## DISCUSSION

The United States (U.S.) has one of the highest rates of overall firearm associated mortality when compared to other developed, high-income countries.^7^,^8^ Most firearm-related injuries and deaths in the U.S. are actually due to suicides and self-inflicted gunshot wounds.^1^,^9^ The rate of firearm associated suicides is 8 times higher in the U.S. when compared to other high-income countries such as Canada and South Korea.^2^ Over the recent decade, the number of suicides has been steadily increasing and is now one of the top 10 leading causes of death in the U.S.^9^ Prior studies have demonstrated that firearm ownership has had a strong association with suicide and intentional self-harm.^10^–^14^

Self-inflicted firearm injury as a form of attempted suicide is more lethal in contrast to other forms of self-inflicted penetrating injury.^6^,^11^–^17^ In spite of the rising rate of attempted suicide and self-inflicted firearm deaths, there has been limited funding to support the research of gun violence. Therefore, it remains difficult to understand the factors and characteristics that contribute to gun violence and suicide.^18^–^19^

Gun ownership is very prevalent in the United States with a population that has the greatest number of civilian-held firearms in the world. It is estimated that there are 265,000,000 to 393,347,000 firearms held by civilians in the United States.^18^,^19^ A recent 2020 poll estimates that 32% of Americans possess a firearm and that 44% live in a households with at least one firearm.^20^

Out of all the firearms that are manufactured and bought in the United States, the handgun is the most popular and most often purchased.^18^,^19^ Overall, firearm ownership has been associated with an increased risk of violent death.^10^–^14^, ^21^, ^23^ Handgun ownership, in particular, appears to be associated with an increased risk of suicide.^10^–^11^, ^21^,^22^ A recent study demonstrates that rates of suicide by any method were higher among handgun owners when compared to non-owners.^18^ A study of suicides in California demonstrated that within the first week after the purchase of a handgun, the rate of suicide among purchasers (644 per 100,000 person-years) was 57 times higher than the adjusted rate of suicide in the general population.^3^ Even in the five years after the legal purchase of a handgun, there is an associated increased risk of suicide.^21^

Similar to previous studies, we found that SIGSW by handgun was associated with increased risk of death and high morbidity when compared to SIGSWs by other gun types. Older, white males with handguns comprise of the highest proportion of suicide by firearm.^10^,^13^,^14^ Prior smaller studies have demonstrated that serious head injuries are often caused by handgun SIGSWs.^14^, ^24^–^26^ A more recent study revealed that SIGSW head and facial injuries had a high survivability, but only in the absence of significant neurological injury.^26^ Those with a GCS 14–15 were likely to have little or no associated brain injury and their wounds were localized to the face. However, SIGSWs that result in brain trauma are significantly associated with mortality.^24^–^26^

Prior to this study, there has been no large, multi-center retrospective analysis on self-inflicted gunshot wound victims who presented to designated trauma centers in the U.S. Our study helps to fill this void by highlighting key characteristics of those persons more likely to die at trauma centers by self-inflicted gunshot wounds.

Our study contributes to the existing literature by examining a large number of trauma patients and documenting the severity of disease, the differing outcomes related to gun type and location of injury, and the incidence of concurrent alcohol and illicit drug use. As expected, SIGSW by HGs led to more lethal conditions with lower GCS scores (less than 9), hypotension, shorter time to death window, and overall higher mortality versus the AOG group overall. Prior smaller studies have demonstrated that illicit drug or alcohol intoxication are implicated in suicide.^25^ A previous study by Bukur et al reported that patients with SIGSWs had a high positivity rate for methamphetamines.^12^ In our cohort, the HG group had a higher prevalence of illicit drug use, while the AOG group had a higher prevalence of alcohol and prescription drug use.

Our results also show that older, White males with handguns pose the highest risk of suicide. Screening and preventative programs should be aimed toward this particular demographic. Because anxiety and depression are common complaints in the ED, routine screening of firearm access and ownership should be performed. A study of eight EDs demonstrated that patients with suicidal ideation or attempts, who had firearms in the home, were not assessed for access to lethal means counseling.^27^ Specifically, asking about hand gun access should be routine, integral part of the history taking of a patient suffering from anxiety, depression, or suicidal ideation. If integrated well into ED treatment plan, lethal means counseling in suicidal patients under 18 years old can be viewed as both favorable and effective. In their interventional study, Runyan and colleagues have found that all of the suicidal youth who were seen in the ED and received lethal means counseling prior to discharge had firearms locked. This is compared to initial 67% of their households reportedly keeping firearms locked prior to the counseling.^28^

Legislative approaches that have been used in limiting firearm access to the general public and have observed decreased incidences of mortality due to SIGSWs. Comprehensive firearm laws such as the National Firearm Agreement (NFA) in Australia limited public firearm ownership in 1996 through regulations and government buy-back program of guns from individual owners.^29^ Several firearm observational studies have found a significant decrease in firearm associated suicides after the passing of the NFA.^29^,^30^

Another legislative approach could would be to expand Gun Violence Restraining Orders (GVROs) as known as “Red Flag Laws” or “Risk Warrants” or “Extreme Risk Protection Order Laws” allow for immediate family members and law enforcement to petition a court to seize and retain firearms from persons who have potential to endanger themselves or others for a finite amount of time. Recently, in California, the GVRO was expanded to include school workers, employers, and co-workers within the last year.^31^–^33^ In San Diego county, there are individual cases that have cited the effectiveness of GVROs actually halting suicides and assault secondary firearms.^33^ It is uncertain as to whether or not the GVROs require physicians, who are otherwise mandatory reporters, to report patients who could be potentially violent (similar to the Tarasoff rule).^33^,^34^ A few studies have found that adopting and enforcing GVROs may lead to an overall decrease firearm suicide.^31^–^35^ Unfortunately, enforcement of GVROs can been variable and some states and jurisdictions may enforce GVROs more heavily than others.^36^,^37^ A recent longitudinal study showed that GVROs could be effective in decreasing suicides in elderly males; the same population that we found to be highest risk in our study.^38^

More prospective studies that can comprehensively compare gun legislation, suicide screening programs, and GVROs as interventions in different regions, counties, states in the U.S should be performed to investigate the effectiveness of these strategies in the prevention of firearm suicides.

## LIMTATIONS

This study provided a large set of the data from multiple, designated trauma centers using ICD-9 codes. The e-codes provided a more reliable set of data than self-reported data. However, there are limitations to using e-codes. Most importantly, data may have been lost or miscoded due human error. Cases are restricted to patients who were seen in the emergency department at a designated trauma center. Therefore, cases of SIGSWs may have been missed due to the fact that the patient did not present to a designated trauma center or may have died prior to arrival to the hospital. Also, the mechanism of injury may not have been known at the of time of ED evaluation and thus not properly e-coded and included within our data.

Additionally, there were 1,405 patients where the weapon type was not clearly identified and another 154 cases that could not be confirmed as SIGSW. As discussed earlier, the intention of the shooter was not completely known and the events leading to the patient’s presentation to the designated trauma center were largely unwitnessed. It cannot be completely known whether or not these the SIGSWs had suicidal intent. The data regarding patients’ toxicology results may also be inaccurate, due to the lack of complete data. Finally, for patients who presented DOA, laboratory evaluation may not have been performed prior to the patient being deceased, creating additional missing data points.

## CONCLUSION

In this large, retrospective study of SIGSWs presenting to designated Trauma Centers in the U.S., handguns were more commonly associated with lethal or near-lethal injuries. Our findings demonstrate that older, White males, who own handguns, are the most at-risk group for lethal and near-lethal SIGSWs. We hope that this study helps demonstrate the crucial need to improve our current gun legislation and to integrate lethal means firearm screening programs in the ED for the most vulnerable patients.

## Figures and Tables

**Table 1:** Comparison of the handgun group vs all other specified gun group (AOG).

Demographics	Handgun (N = 5,139)	All other specified gun (N = 1,130)	P-value
Age			
0–15	78 (2%)	34 (3%)	0.001
16–55	3,821 (74%)	888 (79%)	0.002
>55	1,221 (24%)	202 (18%)	<0.001
Male gender	4,141 (81%)	960 (85%)	0.001
SBP* < 90	1,131 (22%)	190 (17%)	<0.001
Glasgow Coma Scale			
< 9	2,726 (53%)	454 (40%)	<0.001
9–13	209 (4%)	48 (4%)	0.75
14–15	1,986 (39%)	573 (51%)	<0.001
Alcohol present	1,581/3,243 tested (49%)	367/718 tested (51%)	0.25
Drug use**	(2013 tested)	(438 tested)	
No drugs	687 (34%)	150 (34%)	0.96
Illicit drugs	740 (37%)	129 (30%)	0.004
Prescription drugs	719 (36%)	212 (48%)	<0.001

* SBP is systolic blood pressure in millimeters mercury (mmHg) recorded upon hospital arrival.

** Drug use (percent to exceed 100 because many have tested positively to both prescription and illegal drugs).

**Table 2:** Outcomes of head and facial injuries versus those with other body injuries excluding head and face.

	Head or facial injury (N = 4,799)	Other bodily injuries (N = 3,028)	P-value
ED disposition			
Death	1,052 (22%)	111 (4%)	<0.001
ICU	2,768 (58%)	531 (18%)	<0.001
Floor	181 (4%)	660 (22%)	<0.001
OR	536 (11%)	1,303 (43%)	<0.001
Home	107 (2%)	287 (9%)	<0.001
Mortality	2,817 (59%)	365 (12%)	<0.001
Time to death			
DOA (<10 min LOS)	379 (14%)	50 (14%)	0.90
<4 hrs	801 (28%)	213 (58%)	<0.001
4–24 hrs	907 (32%)	38 (10%)	<0.001
24–72 hrs	529 (19%)	6 (2%)	<0.001
>72 hrs	201 (7%)	58 (16%)	<0.001
All body regions injured			
Head	4,114 (86%)	n/a	n/a
Face	2,251 (47%)	n/a	n/a
Neck	252 (5%)	114 (4%)	0.002
Thorax	113 (2%)	1,261 (42%)	<0.001
Abdomen	42 (1%)	885 (29%)	<0.001
Spine	82 (2%)	118 (4%)	<0.001
Upper Extremity	213 (4%)	924 (30%)	<0.001
Lower Extremity	72 (2%)	783 (26%)	<0.001
Unspecified	64 (1%)	96 (3%)	<0.001

*** Drug use (percent to exceed 100 because many tested positively to both prescription and illegal drugs).

*ED*, emergency department; *ICU*, intensive care unit; *OR*, operating room; *DOA*, dead on arrival; *min*, minute; *LOS*, length of stay; *hrs*, hours.
